# Patient-Led Mass Screening for Atrial Fibrillation in the Older Population Using Handheld Electrocardiographic Devices Integrated With a Clinician-Coordinated Remote Central Monitoring System: Protocol for a Randomized Controlled Trial and Process Evaluation

**DOI:** 10.2196/34778

**Published:** 2022-02-01

**Authors:** Kam Cheong Wong, Tu N Nguyen, Simone Marschner, Samual Turnbull, Mason Jenner Burns, Jia Yi Anna Ne, Vishal Gopal, Anupama Balasuriya Indrawansa, Steven A Trankle, Tim Usherwood, Saurabh Kumar, Richard I Lindley, Clara K Chow

**Affiliations:** 1 Westmead Applied Research Centre Faculty of Medicine and Health The University of Sydney Westmead Australia; 2 Westmead Clinical School Faculty of Medicine and Health The University of Sydney Westmead Australia; 3 Bathurst Rural Clinical School School of Medicine Western Sydney University Bathurst Australia; 4 School of Rural Health Faculty of Medicine and Health The University of Sydney Orange Australia; 5 Department of Cardiology Westmead Hospital Westmead Australia; 6 General Practice Department School of Medicine Western Sydney University Campbelltown Australia; 7 The George Institute for Global Health Sydney Australia; 8 Charles Perkins Centre The University of Sydney Sydney Australia

**Keywords:** atrial fibrillation, screening, handheld, electrocardiogram, ECG, acceptability, user perception, user experience, barrier, enabler, older adults, elderly, feasibility, effectiveness, implementation, monitoring, aging, cardiovascular, cardiology, heart disease, mobile phone

## Abstract

**Background:**

Atrial fibrillation (AF) is common in older people and increases the risk of stroke. The feasibility and effectiveness of the implementation of a patient-led AF screening program for older people are unknown.

**Objective:**

This study aims to examine the feasibility and effectiveness of an AF screening program comprising patient-led monitoring of single-lead electrocardiograms (ECGs) with clinician-coordinated central monitoring to diagnose AF among community-dwelling people aged ≥75 years in Australia.

**Methods:**

This is a nationwide randomized controlled implementation trial conducted via the internet and remotely among 200 community-dwelling adults aged ≥75 years with no known AF. Randomization will be performed in a 1:1 allocation ratio for the intervention versus control. Intervention group participants will be enrolled in the monitoring program at randomization. They will receive a handheld single-lead ECG device and training on the self-recording of ECGs on weekdays and submit their ECGs via their smartphones. The control group participants will receive usual care from their general practitioners for the initial 6 months and then commence the 6-month monitoring program. The ECGs will be reviewed centrally by trained personnel. Participants and their general practitioners will be notified of AF and other clinically significant ECG abnormalities.

**Results:**

This study will establish the feasibility and effectiveness of implementing the intervention in this patient population. The primary clinical outcome is the AF detection rate, and the primary feasibility outcome is the patient satisfaction score. Other outcomes include appropriate use of anticoagulant therapy, participant recruitment rate, program engagement (eg, frequency of ECG transmission), agreement in ECG interpretation between the device automatic algorithm and clinicians, the proportion of participants who complete the trial and number of dropouts, and the impact of frailty on feasibility and outcomes. We will conduct a qualitative evaluation to examine the barriers to and acceptability and enablers of implementation. Ethics approval was obtained from the human research ethics committee at the University of Sydney (project number 2020/680). The results will be disseminated via conventional scientiﬁc forums, including peer-reviewed publications and presentations at national and international conferences.

**Conclusions:**

By incorporating an integrated health care approach involving patient empowerment, centralized clinician-coordinated ECG monitoring, and facilitation of primary care and specialist services, it is possible to diagnose and treat AF early to reduce stroke risk. This study will provide new information on how to implement AF screening using digital health technology practicably and feasibly for older and frail populations residing in the community.

**Trial Registration:**

Australian New Zealand Clinical Trials Registry ACTRN12621000184875; https://www.anzctr.org.au/Trial/Registration/TrialReview.aspx?id=380877

**International Registered Report Identifier (IRRID):**

DERR1-10.2196/34778

## Introduction

### Background

The prevalence and incidence of atrial fibrillation (AF) increase with age and is common among older people [1-4]. A recent study estimated that the global prevalence of AF is 59.7 million [4]. Approximately 70% of individuals with AF are aged between 65 and 85 years [5]. AF has been reported to account for 36% of all ischemic strokes, of which 85% are inadequately anticoagulated [6]. If AF is detected early and managed with appropriate anticoagulation therapy, the stroke risk and subsequent stroke-related disability and mortality can be reduced significantly [7]. Unfortunately, it was estimated that 1% of the general population and 1.4% of people aged ≥65 years were living with undiagnosed AF, as reported in a systematic review that combined data from 30 cross-sectional studies (n=122,571) [8]. Hence, the opportunity for anticoagulation therapy to reduce stroke risk for these patients is missed.

Several guidelines have recommended opportunistic screening for AF [9-12]. However, studies suggest that one-off opportunistic screening approaches have a low yield for identifying AF. For example, a recent cluster randomized trial of opportunistic screening using pulse palpation, electronic blood pressure measurement with an AF algorithm, and a handheld single-lead electrocardiogram (ECG) device versus usual care for detection of AF in primary care patients (involving 9218 patients in the intention-to-screen group, 55% women, mean age 75.2 years vs 9526 patients in the usual care group, 54.3% women, mean age 75.0 years) found that opportunistic screening did not improve AF detection compared with usual care [13]. On the contrary, repeated heart rhythm monitoring over a duration increased the yield of AF detection. Petryszyn et al [14] reported in a systematic review that repeated heart rhythm monitoring with ECG devices over periods ranging from 2 weeks to 12 months had higher AF detection rates compared with one-off opportunistic screening approaches: 2.1% (95% CI 1.5-2.8) with repeated ECG screening versus 1.2% (95% CI 0.8-1.6) with opportunistic screening (*P*<.05). Although many guidelines advocate the use of 12-lead ECG for opportunistic screening, these may limit locations where screening may occur and demand a higher skill level to operate 12-lead ECG devices [15,16]. Mobile single-lead handheld ECG devices are easier to use with better time efficiency compared with 12-lead ECG machines, and these single-lead handheld ECG devices have been used in several AF screening studies [15].

Recent systematic reviews, including 8180 single-lead ECG tracings, show that mobile handheld single-lead ECG devices have high accuracy for diagnosing AF [17]. These devices are available to the public and clinicians. However, a national survey reported that although general practitioners (GPs) are aware of the devices, they rarely conduct AF screening in their busy clinical practice [18]. Clinician-led AF screening faces barriers because clinicians are facing competing clinical priorities and time constraints [19]. Alternative strategies for early detection and management of AF are needed. Patient-led AF screening through self-recording of single-lead ECG using mobile handheld ECG devices could be an alternative. A randomized controlled trial (RCT) involving 7173 community-dwelling older people aged 75 to 76 years in Sweden reported that screening using patient-activated intermittent ECG recordings with a handheld ECG device (Zenicor) twice daily over 2 weeks, when the participants noticed palpitations, increased new AF detection fourfold [20]. Similarly, in an RCT of AF screening using a handheld ECG device (AliveCor Kardia) in 1001 participants aged ≥65 years and with CHADS-VASc (congestive heart failure, hypertension, age ≥75 [double score], diabetes, stroke [double score], vascular disease, age 65 to 74 and sex category) score ≥2, the detection rate of new AF after 12 months was 3.8% in the monitored group versus 0.1% in the control group (hazard ratio 3.9, 95% CI 1.4-10.4; *P*=.007) [21]. Another RCT of AF screening using a 2-week ambulatory ECG patch (Zio XT), one at baseline and another at 3 months in 856 participants aged ≥75 years with hypertension, increased new AF detection rate 10 folds (5.3% in the monitored group vs 0.5% in the control group; relative risk: 11.2; 95% CI, 2.7-47.1; *P*=.001) [22]. These studies [20-22] suggest the potential of patient-led approaches to AF screening but still leave questions on how to implement these approaches and the generalizability of these approaches. Unanswered questions about the implementation of AF screening programs include whether such programs can be implemented alongside existing health care systems; whether regular self-screening with mobile devices is feasible and acceptable in older adults; what is the feasibility, resource use, and clinician acceptability of real-world implementation of such programs; what is the time taken by services overseeing and monitoring such programs in terms of reviewing and interpreting large amounts of ECG data; and what strategies can be applied to optimize the use of resources. In addition, there is less data on the barriers to and enablers of the implementation of such programs, longer self-monitoring periods, and implementation in subgroups (such as older people who are frail and people living in remote areas) in which these strategies may not work. A recent systematic review reported that the prevalence of frailty in patients with AF was up to 75% [23]. More studies are needed to better assess whether such mobile health devices can be used effectively and implemented in programs at a large scale among older people who are frail.

There is also a lack of information about the role and importance of patient empowerment with respect to the implementation of AF screening. The World Health Organization promotes patient empowerment (ie, training patients to perform and engage in health-related behaviors within their familiar setting) as it can potentially lead to positive health outcomes [24]. Patient empowerment can be incorporated in patient-led AF screening by training patients to self-record single-lead ECGs. However, patient empowerment has its limitations; that is, patients face automated ECG interpretation results that are often beyond their competence to understand and act upon, and it is impractical to have every ECG result individually and regularly checked by their clinicians. A centralized monitoring system is a feasible way of remotely monitoring a patient’s heart rhythm [25].

The processes of the screening program can vary in their actual implementation because of diverse contexts and participant characteristics (both patients and clinicians); for example, participants may be incapable of or not engage in performing self-recording of ECG, or they may not follow up with (or do not have access to) their clinician after a clinically significant abnormality is detected and notified. Process variations can affect outcomes. Therefore, it is important to evaluate the processes with the aim of better understanding why variations occur and how to improve the processes to achieve an effective intervention and identify contextually relevant strategies to scale up the screening program to benefit larger populations.

**Figure 1:**
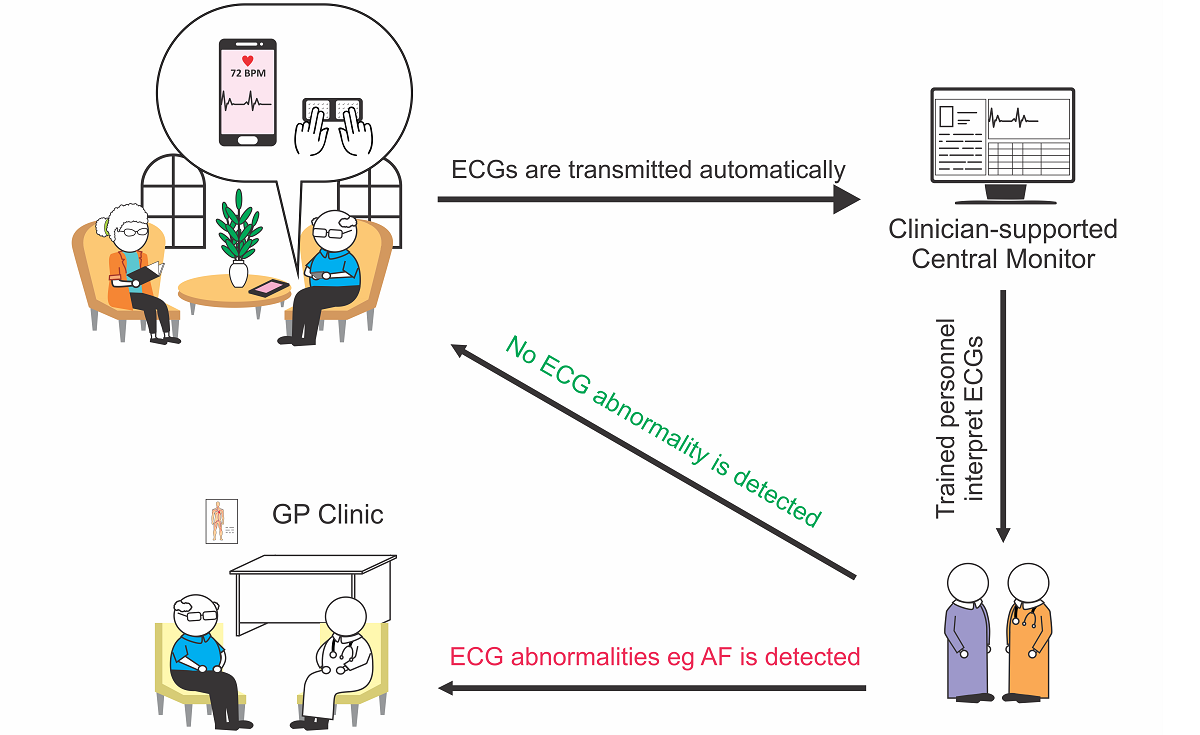
Overview of the *Mass AF* screening program: patient-led self-recording of electrocardiograms (ECGs) with the clinician-coordinated centralized system. AF: atrial fibrillation; GP: general practitioner.

In summary, there are gaps in our knowledge regarding the feasibility, effectiveness, and acceptability of patient-led AF screening by remote patient self-recording with centralized clinician-supported monitoring of single-lead ECGs in older community-dwelling people who are frail. The Mass AF screening program (Figure 1) is designed for implementation among community-dwelling people aged ≥75 years. It comprises the provision of a handheld ECG device and training of participants to self-screen on weekdays and transmit ECGs for review by a central monitoring team. We aim to implement and evaluate this AF self-screening program in which older people in the community are empowered to perform repeated heart rhythm monitoring using a single-lead handheld ECG device and connected with health care providers who review and support the diagnosis of AF and management by primary care and specialist services. We hypothesize that the proposed self-screening model of care may lead to several positive outcomes, including a feasible and scalable model for implementing patient-led AF screening in community-dwelling older people, improved patient satisfaction by empowering them with the relevant knowledge and skills to perform self-screening [24], and thereby higher adherence to the screening program [26,27].

### Objective

Our study objectives are to (1) compare AF ascertainment rates in the intervention and control groups; (2) evaluate the feasibility of the intervention, including assessing participant satisfaction, acceptability, barriers, and enablers and how frailty influences these assessments; and (3) assess agreements between the ECG device automatic algorithm and clinician interpretation.

Alongside these objectives, the specific objectives of the process evaluations are as follows:

1. To assess the fidelity of the screening program (ie, whether the intervention was delivered as intended), participant engagement with the intervention in terms of the frequency of ECG recordings, and reach (eg, the socioeconomic and frailty profiles of participants and how these profiles affect the engagement and outcomes)
2. To evaluate the feasibility of the screening program from the perspective of participants and clinicians to gain a deeper understanding of barriers and enablers; this includes an examination of the mechanisms of impact; that is, an examination of the potential causal mechanisms through which the intervention results in the adoption of self-screening by understanding how patients and clinicians interact with the screening program
3. To explore any factors external to the screening program that may have affected implementation (ie, the community-dwelling environment, access to health care services, and GP views and attitudes), including identification of resources and implementation processes required for effective uptake and implementation of the screening program

## Methods

### Study Design

**Figure 2:**
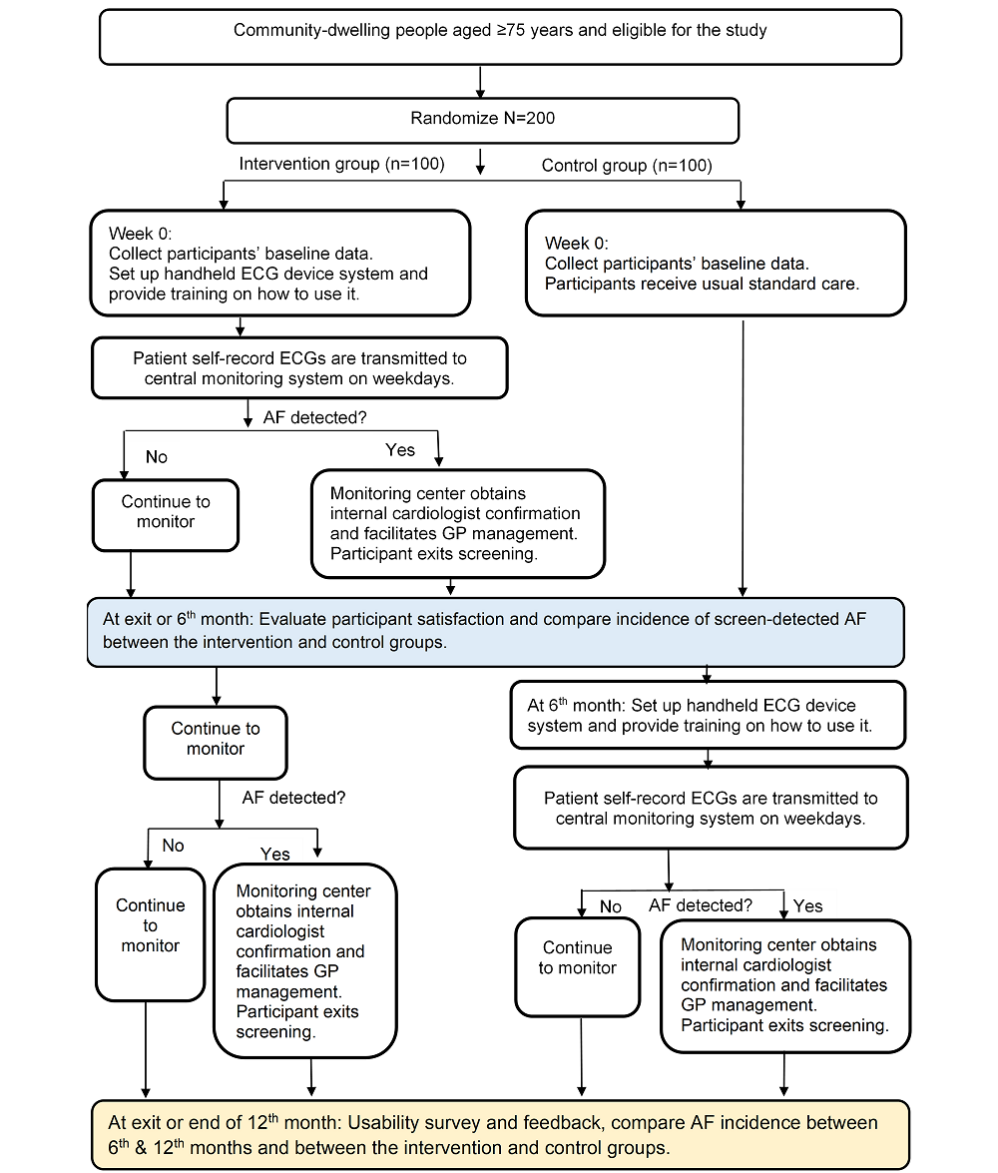
Flowchart of the *Mass AF* screening program. AF: atrial fibrillation; ECG: electrocardiogram; GP: general practitioner.

This is a 2-arm, randomized, open-label, waitlist-controlled trial in community-dwelling people aged ≥75 years. We will also conduct a process evaluation of this study. Randomization in the ratio of 1:1 is stratified by participant frailty status (frail or nonfrail; Multimedia Appendix 1). Participant frailty was determined using the FRAIL (Fatigue, Resistance, Ambulation, Illnesses, and Loss of Weight) scale based on five components: *fatigue*, *resistance* (inability to climb stairs), *ambulation* (inability to walk a certain distance), *illness*, and *loss of weight* [28,29]. The intervention group will commence the monitoring program for 12 months upon enrollment. The control group will be waitlisted for the first 6 months and then commence the monitoring program in the subsequent 6 months. The steps involved for enrollment, randomization, intervention, control, and exit from the program are outlined in the study flowchart (Figure 2), and descriptions of the screening program are provided in the following sections.

### Study Population

Our target population involves older people living independently in the community outside of a hospital, nursing home, or similar institutional residence. The inclusion criteria are as follows: community-dwelling people aged ≥75 years, having a smartphone or electronic device that can operate the AliveCor Kardia mobile app, and being able to understand instructions in English. Individuals with the following conditions will be excluded: previously confirmed diagnosis of AF, having an implantable cardiac monitor, pacemaker or defibrillator, dementia, inability to provide informed consent, and those with a medical illness with an anticipated life expectancy of <3 months.

### Intervention Group: AF Self-screening and Monitoring Program

Participants allocated to the intervention will immediately commence the monitoring program. They will be provided with a small handheld single-lead Kardia ECG device (AliveCor Inc), which has been cleared by the Food and Drug Administration and approved by the Therapeutics Goods Administration. After the participants receive the device, they will receive a phone call from research assistants who will help them to set up the device, including downloading the Kardia app to the mobile phone, setting up reminders to record ECG in the app, and setting up a Kardia user account. The research assistants will create a participant profile in the central monitoring portal, which will generate a unique 12-digit code for the participant. Research assistants will inform participants of their unique 12-digit code via SMS text message or over the phone. The participant will enter the 12-digit code in their Kardia user account, and once this step is completed, the Kardia user account will be connected to the central monitoring portal. Participants can commence recording the ECGs, which will be transmitted to their personal profiles in the central monitoring portal.

Research assistants will take participants through the steps of recording an ECG. Participants will record a single-lead ECG trace by placing 2 fingers of each hand steadily on 2 small touchpads (3 cm × 3 cm) of the device for 30 seconds on weekdays. The ECG device will be connected to the mobile phone wirelessly via the Kardia app that they have downloaded. An ECG trace will appear on the participants’ mobile phones, and participants will be able to record notes in the ECG trace. We will encourage them to note the activities they performed before recording the ECG. The ECG and notes will be automatically transmitted to the central monitoring portal. The training conducted over the telephone or video call between research assistants and participants will take approximately 30 to 60 minutes per participant. In the context of the COVID-19 pandemic, all study-related procedures will be conducted remotely using phone calls or video calls (if participants have access to and prefer this modality). To ensure that participants are confident in using the device, research assistants will call each participant to confirm that they are able to record an ECG. Participants will be encouraged to have a family member who may assist them in the process. After the training, participants will also receive an SMS text message with a weblink to an instructional video created by the device manufacturer. The short training video will serve as a reference for participants to refresh their memory on how to record a single-lead ECG. The research team will contact participants if they have not recorded any ECGs for 3 consecutive days to find out and address the causes, if possible.

Before commencing the study, participants will be advised that in the event of experiencing symptoms (eg, syncope, chest pain, palpitations, and shortness of breath) that are severe in nature or that are of concern to the participants, to present to their local medical physician or hospital for assessment as soon as possible.

### Control Group: Usual Care During Waiting Period

Participants allocated to the control group will have usual care and be told that they have been waitlisted to start the monitoring program in 6 months. During the 6-month waiting period, it is expected that participants in the waitlist group will visit their GPs as per their usual health care needs, and their GPs will provide care and referrals as usual.

### Sample Size

In computing the sample size required to assess the primary feasibility outcome, we will evaluate the proportion of participants reporting being satisfied or very satisfied that their heart rhythm was monitored in the past 6 months in the intervention group versus the control group. We arbitrarily set that 50% of the participants in the control group will be satisfied or very satisfied. With reference to the literature that reported a proportion of 67% [30] to 82% [31] of older people were satisfied or very satisfied with the use of technology-enabled monitoring at home, we postulate that there will be an absolute 30% increase in satisfaction in the intervention group compared with the control group. Our study will have 80% power, using a 5% level of significance, to detect an absolute difference of 30% in satisfaction between the 2 groups. A sample size of 100 participants aged ≥75 years is required to assess the primary feasibility outcome

To calculate the sample size required to evaluate the primary clinical outcome of AF detection rate, we set an AF detection rate of 10% in the intervention group and 1% in the control group, in accordance with a recent study [22]. At 80% power, a 2-sided test, and α .05, we estimate that a sample of 200 participants will be needed to detect a significant difference in AF detection between the intervention and control groups. Therefore, a total of 200 participants will be recruited for this trial to assess the primary clinical outcome.

### Randomization

Participants will be randomized to the intervention or control group on a 1:1 basis stratified by baseline measure of frailty (ie, frail or not frail according to the FRAIL score) [28,29] and using permuted blocks of sizes 4 and 6. The statistician has generated a randomization list using the RandomiseR package in R software (R Foundation for Statistical Computing) [32]. The randomization list will be input into the REDCap (Research Electronic Data Capture) [33] database, which captures participant demographic and baseline data and survey findings (Multimedia Appendix 1). The statistician and principal investigators will be unaware of patient allocation until after the completion of the study.

### Recruitment

A multipronged approach will be used to identify potential participants. We will use clinician networks (eg, GPs, cardiologists, geriatricians, and allied health professionals) and a variety of direct approaches to the community to recruit a wide spectrum of participants from various demographic backgrounds living in wide geographical areas across Australia. Communications will be sent to practice managers seeking their assistance in disseminating introductory letters, leaflets, flyers, and posters to their clinicians. The practice managers may also disseminate the information to their patients through their usual channels of communication, including displaying them in waiting rooms, websites, newsletters, or in electronic format or hard copy. The decision to contact the research team will be at the discretion of the patients. In addition, a direct community approach will be used. The research poster and leaflets will be disseminated in local community centers such as the Returned and Services League Australia and places of worship. People interested in the study will initiate contact with the study team directly by email or phone to receive further information. We will also list the study with third-party recruiters such as HealthMatch [34] and Join US [35]. Individuals who contact the research team will be screened for eligibility and provided with further explanations about the study. We will inform GPs about their patients who enroll in the study.

### Participant Consent and Enrollment

The research team will confirm the eligibility of interested individuals against the inclusion and exclusion criteria over the phone. Eligible individuals will be provided with participant information statements and consent information and given time to read the information. Research personnel will answer individuals’ questions. Participants will provide verbal consent to a member of the research team who will electronically sign off the consent form and keep the form in the secured university computer drive.

### ECG Central Monitoring System

Qualified and trained study personnel, including a cardiac technician and clinical monitoring personnel with medical qualifications, will remotely review all ECGs and compare their diagnosis with the device’s automated diagnostic algorithm. If the ECGs are normal or have minimal abnormalities and the personnel are certain of their diagnosis, the ECGs will not be referred to a cardiologist. However, the personnel will refer all abnormal ECGs for diagnosis confirmation, or uncertain ECG abnormalities for clarification of diagnosis, to cardiologists or cardiac electrophysiology specialists. Participants’ ECGs will be classified into *low, moderate, high, and severe abnormalities* and managed as shown in Table 1. The research team will notify the participants and their GPs of AF or other clinically significant ECG abnormalities. A copy of an abnormal ECG will be forwarded to their GP. When an AF diagnosis is confirmed by the research team, the participant will be advised to see their GP. The participant will exit the screening program or opt to continue the monitoring program. The research team will contact the GP to obtain information about the treatment given to the patient.

**Table 1:** Electrocardiogram (ECG) classification and management plan.

ECG findings	Classification	The study team will take the following actions
First-degree heart block	Low critical abnormality	If PR interval >300 milliseconds, notify and send ECG to GP^a^ within a week If PR interval is between 201 and 300 milliseconds, notify and send ECG to GP within the duration of participant’s enrollment in the study
Ectopic heartbeats (atrial ectopic and ventricular ectopic)	Low critical abnormality	As these are common and noncritical findings, notify GP at the end of the study
Atrial fibrillation, atrial flutter, nonsustained ventricular tachycardia, bradycardia <40 bpm^b^, second-degree heart block, nonsustained supraventricular tachycardia	Moderate critical abnormality	Notify and send ECG to GP within a week Advise patients to see their GP as soon as possible Contact patient to confirm review with their GP in the subsequent week
Significant ECG abnormalities that need urgent medical attention (eg, suspected ST elevation)	High critical abnormality	Consult cardiologists in the research team to confirm the diagnosis, and where necessary, adjudicate suspect ECGs Notify and send ECG to GP within 3 working days Advise patients to see their GP as soon as possible Contact patient to confirm review with their GP in the subsequent week
Potentially life-threatening arrhythmia or abnormality (eg, third-degree heart block)	Severe abnormality	Consult cardiologists in the research team to confirm diagnosis Advise patients to present to their local emergency department immediately Notify and send ECG to GP on the same day Contact patient to confirm review with their GP in the subsequent week
ECGs without any of the above abnormalities	Normal	Review ECG report (including normal and the above abnormal findings) in monthly team meeting

^a^GP: general practitioner.

^b^bpm: beats per minute.

### Data Collection

All study procedures have been designed to be conducted remotely using telephone or video calls. At baseline, we will obtain information on sociodemographics, self-reported weight and height, and concurrent medical conditions and medications and data to assess stroke risk, frailty, and activities of daily living. At the end of the program, we will conduct a usability survey of all participants via phone calls to obtain information related to their experiences with the screening program and obtain further information on any adverse events while participating in the program (Multimedia Appendix 1). All GPs who have patients enrolled in the study will be invited to provide their feedback in a survey (Multimedia Appendix 2). All electronic data and documents related to participants and the project will be securely stored in the university computer drive accessible by authorized research team members only.

### Qualitative Evaluations

We have followed the Medical Research Council guidelines in designing the process evaluation [36]. Participants’ and GPs’ expectations and experiences of the screening program may be influenced by various contextual factors such as the participants’ social and cultural background and the GPs’ clinical practice resource and setting. Using a theoretical lens of critical realism [37], we will provide an explanatory analysis of the perceptions, experiences, and interactions with contextual factors that participants describe (ie, what works for whom and under what circumstances).

After participants have used the handheld ECG device for a minimum of 3 months, the research team will invite several participants in both groups (≥10 participants to achieve thematic saturation) to attend an in-depth semistructured interview to explore their views and feedback on the study. The semistructured interview will explore the participants’ feedback on their experiences with using the ECG device, use of the device for detecting irregular heart rhythm, participants’ perceptions of this remote screening method, and their access to health services generally (Multimedia Appendix 1). The invitation will be based on purposive sampling to obtain representative participants from a wide spectrum of demographic groups, that is, male or female, rural or urban, and frail or not frail.

GPs will be invited to a one-on-one in-depth interview, which will take approximately 20 to 30 minutes. The interviews will be audio recorded and transcribed by the research team. We plan to recruit at least eight GPs for this interview. A sample of 8 GPs is considered appropriate for the exploratory interviews [38]. However, more GPs will be recruited if thematic saturation is not achieved. The semistructured interviews will explore GPs’ views with respect to AF screening generally and in screening people aged ≥75 years particularly, their views of the patient-led self-screening in this research study, their knowledge of the use of mobile health devices (including handheld ECG devices) in clinical practice, and their views on using the handheld ECG device (ie, AliveCor Kardia) for AF screening in this study. In alignment with a critical realism [37] approach, the interview questions will explore GPs’ contextual factors and their interaction with patient participants and the research team with the aim of exploring GPs’ perception of their roles and barriers to and acceptability and enablers of this patient-led self-recording of a single-lead ECG program (Multimedia Appendix 2).

### Outcome Measurements

The clinical outcomes include (1) new AF detected over 6 and 12 months and (2) appropriate use of anticoagulant therapy. The feasibility outcomes include (1) participant satisfaction that their heart rhythm was monitored in the past 6 months; (2) participant recruitment rate; (3) frequency of ECG transmission to the central monitor; (4) proportion of participants who complete the program; (5) proportion of dropouts (exit the program prematurely) and reasons; (6) actual costs of the screening program; (6) agreement between the ECG device’s automatic algorithm and clinician interpretation; (7) usability assessment; (8) participant acceptability and barriers to and enablers of implementation; and (9) impact of frailty on feasibility assessments and outcomes.

### Analysis

All analyses will be conducted according to the principle of intention-to-treat. Continuous variables will be presented as mean (SD) or median with IQR and categorical variables as frequency and percentage. The key outcomes, namely participant satisfaction in the AF screening program, usability of the screening program, and the incidence of new AF detected over 6 and 12 months, will be reported as frequency and percentages and will be compared between the intervention and control groups using the chi-square test. Comparisons between groups (intervention and control) for other outcomes will be assessed using the chi-square test or Fisher exact test for categorical variables and Student *t* test or Mann-Whitney test for continuous variables as appropriate. We will consider 2-tailed *P*<.05 as statistically significant. The agreement between the ECG device automatic algorithm and clinician interpretation will be evaluated using κ statistics. Subgroup analyses will be performed based on age, gender, and location. Subgroup analysis by frailty will be performed to examine the potential impact of frailty on the outcomes, feasibility, and acceptability of the program. The actual operational costs of delivering the project will be recorded and verified using invoices and receipts, including the costs of personnel involved in interpreting ECGs, which will be computed based on their hourly wages and the time they spent in their roles in this program. The resultant costs will be compared with the costs reported in the literature on the costs associated with the detection of AF in a similar older population. Missing data will be identified, and its causes will be described. Sensitivity analysis will be performed to examine the robustness of the findings [39].

Qualitative evaluation will be reported according to the COREQ (Consolidated Criteria for Reporting Qualitative Research) guidelines [40]. Interviews with patients and GPs will be thematically analyzed using an inductive approach [41]. Themes will be interpreted through the critical realism lens [37] and compared with the literature [42,43]. We will triangulate the quantitative and qualitative findings [44] from patient participants and GPs to acquire an in-depth understanding of the barriers to and enablers of implementing the screening program.

The process evaluation components [36], explanatory data, and anticipated outcomes are summarized in Table 2.

The quantitative and qualitative analysis approach and methods are summarized in Textbox 1.

**Table 2:** Process evaluation—implementation processes, mechanisms of impact, and contexts.

Process evaluation components	Descriptions	Methods and explanatory data	Anticipated outcomes
Implementation processes	Fidelity of implementation Participation in intervention Reach	Participant enrollment and characteristics, including socioeconomic status and frailty Participant engagement (number of self-recorded ECGs^a^) Clinician characteristics and involvement	Participants who are engaged with the intervention and satisfied with the program
Mechanisms of impact (how does intervention help adoption of AF^b^ self-screening)	Barriers and enablers	Participant engagement and satisfaction Participant survey and interview Clinician survey and interview	A feasible screening program
Context (how do factors external to the intervention affect uptake and implementation)	Participants’ overall health Community-dwelling environment Access to health care services General practitioner views and attitudes	Comorbidities, frailty, and functional status Participant demographic data, survey, and interview Clinician survey and interview	Identification of resources and implementation processes required for effective uptake and implementation of the screening program A contextualized feasible screening program

^a^ECG: electrocardiogram.

^b^AF: atrial fibrillation.

**Description of analysis methods on outcome measures compared between the intervention group and waitlist control group.**

**New atrial fibrillation detected**

- Frequency of occurrence and proportions at 6 months—via electrocardiogram collected at the central monitor (during intervention) and self-report and confirmation with medical records or electrocardiogram (during waitlist control period)

**Appropriate use of anticoagulant therapy**

- Frequency of occurrence and proportion of participants with new atrial fibrillation treated with anticoagulant appropriately—via confirmation with general practitioners or participants’ self-reported anticoagulant medication use assessed by using an interviewer-administered questionnaire at 6 months

**Participant satisfaction at the sixth month**

- Frequency of occurrence and proportion of participants reporting being satisfied or very satisfied—assessed via an interviewer-administered questionnaire at 6 months

**Participant recruitment rate**

- Number of participants recruited over time—via log sheet
- Cumulative frequency graph over time

**Electrocardiogram transmission to the central monitor**

- Frequency of electrocardiogram transmission per participant over the enrollment period—electronic logs of all transmissions to the central monitor
- The time the participant transmitted the electrocardiogram—histogram of electrocardiogram transmission time distribution

**Participants who completed the program**

- Number and proportion of participants who completed the program—via log sheet

**Proportion of dropouts (exit program prematurely) and reasons**

- Number and proportion of dropouts and reasons—via log sheet

**Actual costs of the screening program**

- Operational costs (eg, electrocardiogram devices, subscription fee to Kardia monitoring portal, and mail postages) recorded and verified using invoices and receipts
- Costs of personnel involved in interpreting electrocardiograms computed based on their hourly wage and the time they spent in their roles in this program—data collected prospectively and throughout program implementation

**Agreement in electrocardiogram interpretations**

- Number of consultations and percentages of agreement between the monitoring personnel and cardiologists in clarifying uncertain electrocardiogram abnormalities—logs of all interactions

**Usability assessment at the 12th month**

- Responses to the Usability questionnaire will be assessed by 5-point Likert scale—via self-report questionnaires (Multimedia Appendix 1)

**Participant acceptability and barriers to and enablers of implementation**

- In-depth one-on-one interview with participants (Multimedia Appendix 1) and general practitioners (Multimedia Appendix 2)
- Thematic analysis

**Impact of frailty on feasibility assessments and the outcomes**

- Frailty assessed by the 5-item FRAIL (Fatigue, Resistance, Ambulation, Illnesses, and Loss of Weight) scale (Multimedia Appendix 1)

### Ethics and Dissemination

This study was approved by the human research ethics committee of the University of Sydney (reference number 2020/680). The study is conducted in full conformance with principles of the International Committee on Harmonization of Good Clinical Practice and *Declaration of Helsinki* Good Clinical Practice guidelines and within the laws and regulations of the Australian National Health and Medical Research Council.

## Results

This study was funded by a National Heart Foundation Vanguard grant awarded in October 2019. The study was approved by the human research ethics committee of the University of Sydney in November 2020. The first participant was enrolled in May 2021. As of December 2021, a total of 112 participants have been enrolled. Data analysis and results are expected to be published in December 2023.

## Discussion

### Anticipated Strengths

This patient-led AF screening in the community is different from clinician-led opportunistic screening. In this model of screening, participants are trained and empowered to self-record ECGs instead of awaiting clinicians to screen them opportunistically. The centralized remote monitoring team will facilitate patient access to see their GPs.

Drawing on the strengths of quantitative and qualitative methodologies [45], this study will provide evidence for AF detection rates, participant satisfaction, and feasibility of implementing this program using a telephone, a video interface, and the internet for older people, including people who are frail, with the potential to extend to other vulnerable groups such as people with disabilities, people who are socially isolated or because of the COVID-19 pandemic lockdown, and those who live in remote areas.

Participant satisfaction scores often reflect the convergence and gap between participant expectations and actual experiences [46], and satisfaction scores also measure how well the intervention was received by participants [31]; hence, satisfaction is a commonly evaluated outcome in clinical trials [46]. Nonetheless, satisfaction scores would not provide insights into participant experiences, which provide contextualized feedback to improve the screening program. We complement this with in-depth one-on-one semistructured interviews with participants and GPs to explore their insights on barriers and enablers.

This is a prospective RCT design. The waitlist-controlled design provides equitable access to all participants in a mass screening strategy. It is a simple, acceptable, and noninvasive screening strategy that can be implemented regardless of geographical location. Our novel approach in promoting patient-empowered self-screening integrated with a clinician-coordinated centralized system will provide patients with integrated care that facilitates access to GPs and specialist services. Patients will receive training to use the device, and they will be reminded to perform their routine ECGs if they have not done so for 3 consecutive days. This type of interaction and reminder system has been proven to yield positive health outcomes such as engagement in positive health behaviors [47]. Performing self-screening could raise awareness of self-care and improve patient confidence in self-care, which is a form of patient empowerment that is promoted by the World Health Organization [24].

We anticipate that this study will provide data on whether implementation of this type of community-based model of care is feasible and acceptable to patients and health providers in the community. At study completion, the results will be shared with the Heart Foundation (study funder), policy makers, health providers, consumers, and other stakeholders. Access to ECG monitoring devices for future screening programs is dependent on feasibility from a cost perspective, as well as aspects of whether this would be a barrier to implementation. We will conduct a qualitative analysis to understand participants’ perceptions of the value of the monitoring device to their well-being, as well as the affordability of the device.

### Anticipated Research Outcomes and Impacts on Clinical Practice and Policy

This study will provide information on the usability of and costs associated with AF mass screening in Australian people aged ≥75 years. It will also provide evidence of AF incidence in older people in Australia. This can potentially facilitate the development of a national screening program for AF in older people and people who are frail.

Screening for AF is more likely to occur in the community or general practice setting than in the hospital setting. It took an average of 10.6 minutes to acquire a 12-lead ECG in a general practice setting (including the time preparing the patient for ECG acquisition and placing the electrodes correctly on the patient) [48]. In contrast, this patient-empowered self-screening potentially reduces time constraints faced by clinicians as patients are empowered to self-record ECGs in the community rather than clinicians spending the additional time acquiring ECGs opportunistically in a busy clinical setting. In this program, GPs can access help from the participating cardiology team to confirm the diagnosis and facilitate appropriate management of new AF if necessary. This could enhance access and interaction between GPs and cardiologists in providing integrated care to patients to achieve better health outcomes.

### Anticipated Challenges and Limitations

All study procedures have been designed to be conducted remotely by telephone or video interface to facilitate this study in the context of the COVID-19 pandemic. Therefore, the collection of baseline data and medical history relies on participants’ self-reported information. There is a potential for loss to follow-up in older patients who are frail; for example, participants do not record ECGs. We will attempt to minimize this by following up with patients when no ECG is received for 3 consecutive days. There may be a potential selection bias in this study based on the inclusion criteria. For example, this trial is limited to older people who can understand English and have a smartphone. These participants may come from higher socioeconomic communities and represent a more motivated cohort than the general population of older people.

### Conclusions

The findings from this implementation study will guide the development of practical and attainable solutions to address a gap in AF screening among older people in the community and other vulnerable groups. In addition, this study will explore the experiences and feedback from participants and clinicians and provide new knowledge on the processes involved in the implementation of the screening program and how processes can be improved, replicated, and scaled up to reach larger populations.

## Appendix

Multimedia Appendix 1 Patient participant data collection at baseline, 6-month follow-up, and 12-month completion of the program and semistructured interview guide.

Multimedia Appendix 2 General practitioner data form and semistructured interview guide.

## Abbreviations

AF: atrial fibrillation
CHADS-VASc: congestive heart failure, hypertension, age ≥75 (double score), diabetes, stroke (double score), vascular disease, age 65 to 74 and sex category
COREQ: Consolidated Criteria for Reporting Qualitative Research
ECG: electrocardiogram
FRAIL: Fatigue, Resistance, Ambulation, Illnesses, and Loss of Weight
GP: general practitioner
RCT: randomized controlled trial
REDCap: Research Electronic Data Capture

## References

[1] Andrade JG, Macle L, Nattel S, Verma A, Cairns J (2017). Contemporary Atrial Fibrillation Management: a comparison of the current AHA/ACC/HRS, CCS, and ESC guidelines. Can J Cardiol.

[2] Ball J, Thompson DR, Ski CF, Carrington MJ, Gerber T, Stewart S (2015). Estimating the current and future prevalence of atrial fibrillation in the Australian adult population. Med J Aust.

[3] Heeringa J, van der Kuip DA, Hofman A, Kors JA, van Herpen G, Stricker BH, Stijnen T, Lip GY, Witteman JC (2006). Prevalence, incidence and lifetime risk of atrial fibrillation: the Rotterdam study. Eur Heart J.

[4] Roth GA, Mensah GA, Johnson CO, Addolorato G, Ammirati E, Baddour LM, Barengo NC, Beaton AZ, Benjamin EJ, Benziger CP, Bonny A, Brauer M, Brodmann M, Cahill TJ, Carapetis J, Catapano AL, Chugh SS, Cooper LT, Coresh J, Criqui M, DeCleene N, Eagle KA, Emmons-Bell S, Feigin VL, Fernández-Solà J, Fowkes G, Gakidou E, Grundy SM, He FJ, Howard G, Hu F, Inker L, Karthikeyan G, Kassebaum N, Koroshetz W, Lavie C, Lloyd-Jones D, Lu HS, Mirijello A, Temesgen AM, Mokdad A, Moran AE, Muntner P, Narula J, Neal B, Ntsekhe M, de Oliveira GM, Otto C, Owolabi M, Pratt M, Rajagopalan S, Reitsma M, Ribeiro AL, Rigotti N, Rodgers A, Sable C, Shakil S, Sliwa-Hahnle K, Stark B, Sundström J, Timpel P, Tleyjeh IM, Valgimigli M, Vos T, Whelton PK, Yacoub M, Zuhlke L, Murray C, Fuster V, GBD-NHLBI-JACC Global Burden of Cardiovascular Diseases Writing Group (2020). Global burden of cardiovascular diseases and risk factors, 1990-2019: update from the GBD 2019 study. J Am Coll Cardiol.

[5] Feinberg W, Blackshear JL, Laupacis A, Kronmal R, Hart RG (1995). Prevalence, age distribution, and gender of patients with atrial fibrillation. Analysis and implications. Arch Intern Med.

[6] Leyden JM, Kleinig TJ, Newbury J, Castle S, Cranefield J, Anderson CS, Crotty M, Whitford D, Jannes J, Lee A, Greenhill J (2013). Adelaide stroke incidence study. Stroke.

[7] You JJ, Singer DE, Howard PA, Lane DA, Eckman MH, Fang MC, Hylek EM, Schulman S, Go AS, Hughes M, Spencer FA, Manning WJ, Halperin JL, Lip GY (2012). Antithrombotic therapy for atrial fibrillation: Antithrombotic Therapy and Prevention of Thrombosis, 9th ed: American College of Chest Physicians Evidence-Based Clinical Practice Guidelines. Chest.

[8] Lowres N, Neubeck L, Redfern J, Freedman SB (2013). Screening to identify unknown atrial fibrillation. A systematic review. Thromb Haemost.

[9] Brieger D, Amerena J, Attia J, Bajorek B, Chan KH, Connell C, Freedman B, Ferguson C, Hall T, Haqqani H, Hendriks J, Hespe C, Hung J, Kalman JM, Sanders P, Worthington J, Yan TD, Zwar N, NHFA CSANZ Atrial Fibrillation Guideline Working Group (2018). National Heart Foundation of Australia and the Cardiac Society of Australia and New Zealand: Australian Clinical Guidelines for the Diagnosis and Management of Atrial Fibrillation 2018. Heart Lung Circ.

[10] Freedman B, Camm J, Calkins H, Healey JS, Rosenqvist M, Wang J, Albert CM, Anderson CS, Antoniou S, Benjamin EJ, Boriani G, Brachmann J, Brandes A, Chao TF, Conen D, Engdahl J, Fauchier L, Fitzmaurice DA, Friberg L, Gersh BJ, Gladstone DJ, Glotzer TV, Gwynne K, Hankey GJ, Harbison J, Hillis GS, Hills MT, Kamel H, Kirchhof P, Kowey PR, Krieger D, Lee VW, Levin LÅ, Lip GY, Lobban T, Lowres N, Mairesse GH, Martinez C, Neubeck L, Orchard J, Piccini JP, Poppe K, Potpara TS, Puererfellner H, Rienstra M, Sandhu RK, Schnabel RB, Siu CW, Steinhubl S, Svendsen JH, Svennberg E, Themistoclakis S, Tieleman RG, Turakhia MP, Tveit A, Uittenbogaart SB, Van Gelder IsC, Verma Atul, Wachter Rolf, Yan BP, AF-Screen Collaborators (2017). Screening for atrial fibrillation: a report of the AF-SCREEN International Collaboration. Circulation.

[11] Kirchhof P, Benussi S, Kotecha D, Ahlsson A, Atar D, Casadei B, Castella M, Diener HC, Heidbuchel H, Hendriks J, Hindricks G, Manolis AS, Oldgren J, Popescu BA, Schotten U, Van Putte B, Vardas P, Agewall S, Camm J, Esquivias GB, Budts W, Carerj S, Casselman F, Coca A, De Caterina R, Deftereos S, Dobrev D, Ferro JM, Filippatos G, Fitzsimons D, Gorenek B, Guenoun M, Hohnloser SH, Kolh P, Lip GY, Manolis A, McMurray J, Ponikowski P, Rosenhek R, Ruschitzka F, Savelieva I, Sharma S, Suwalski P, Tamargo JL, Taylor CJ, Van Gelder IC, Voors AA, Windecker S, Zamorano JL, Zeppenfeld K (2016). 2016 ESC Guidelines for the management of atrial fibrillation developed in collaboration with EACTS. Eur J Cardiothorac Surg.

[12] Mairesse G, Moran P, Van Gelder IC, Elsner C, Rosenqvist M, Mant J, Banerjee A, Gorenek B, Brachmann J, Varma N, de Lima GG, Kalman J, Claes N, Lobban T, Lane D, Lip GY, Boriani G, ESC Scientific Document Group (2017). Screening for atrial fibrillation: a European Heart Rhythm Association (EHRA) consensus document endorsed by the Heart Rhythm Society (HRS), Asia Pacific Heart Rhythm Society (APHRS), and Sociedad Latinoamericana de Estimulación Cardíaca y Electrofisiología (SOLAECE). Europace.

[13] Uittenbogaart SB, van Gurp NV, Lucassen WA, Winkens B, Nielen M, Erkens PM, Knottnerus JA, van Weert HC, Stoffers HE (2020). Opportunistic screening versus usual care for detection of atrial fibrillation in primary care: cluster randomised controlled trial. Br Med J.

[14] Petryszyn P, Niewinski P, Staniak A, Piotrowski P, Well A, Well M, Jeskowiak I, Lip G, Ponikowski P (2019). Effectiveness of screening for atrial fibrillation and its determinants. A meta-analysis. PLoS One.

[15] Walker A, Muhlestein J (2018). Smartphone electrocardiogram monitoring: current perspectives. Adv Health Care Technol.

[16] Wong K, Thiagalingam A, Kumar S, Marschner S, Kunwar R, Bailey J, Kok C, Usherwood T, Chow CK (2021). User perceptions and experiences of a handheld 12-lead electrocardiographic device in a clinical setting: usability evaluation. JMIR Cardio.

[17] Wong KC, Klimis H, Lowres N, von Huben A, Marschner S, Chow CK (2020). Diagnostic accuracy of handheld electrocardiogram devices in detecting atrial fibrillation in adults in community versus hospital settings: a systematic review and meta-analysis. Heart.

[18] Wong KC, Kok C, Marschner S, Usherwood T, Chow CK (2020). Screening for atrial fibrillation and other arrhythmias in primary care. BMC Fam Pract.

[19] Orchard J, Li J, Freedman B, Webster R, Salkeld G, Hespe C, Gallagher R, Patel A, Kamel B, Neubeck L, Lowres N (2020). Atrial fibrillation screen, management, and guideline-recommended therapy in the rural primary care setting: a cross-sectional study and cost-effectiveness analysis of ehealth tools to support all stages of screening. J Am Heart Assoc.

[20] Svennberg E, Engdahl J, Al-Khalili F, Friberg L, Frykman V, Rosenqvist M (2015). Mass screening for untreated atrial fibrillation: the STROKESTOP study. Circulation.

[21] Halcox JP, Wareham K, Cardew A, Gilmore M, Barry JP, Phillips C, Gravenor MB (2017). Assessment of remote heart rhythm sampling using the AliveCor heart monitor to screen for atrial fibrillation: the REHEARSE-AF study. Circulation.

[22] Gladstone DJ, Wachter R, Schmalstieg-Bahr K, Quinn FR, Hummers E, Ivers N, Marsden T, Thornton A, Djuric A, Suerbaum J, von Grünhagen D, McIntyre WF, Benz AP, Wong JA, Merali F, Henein S, Nichol C, Connolly SJ, Healey JS, SCREEN-AF Investigators and Coordinators (2021). Screening for atrial fibrillation in the older population: a randomized clinical trial. JAMA Cardiol.

[23] Villani ER, Tummolo AM, Palmer K, Gravina EM, Vetrano DL, Bernabei R, Onder G, Acampora N (2018). Frailty and atrial fibrillation: a systematic review. Eur J Intern Med.

[24] (2009). Patient empowerment and health care. WHO Guidelines on Hand Hygiene in Health Care: First Global Patient Safety Challenge Clean Care Is Safer Care.

[25] Health Quality Ontario (2018). Remote monitoring of implantable cardioverter-defibrillators, cardiac resynchronization therapy and permanent pacemakers: a health technology assessment. Ont Health Technol Assess Ser.

[26] Fuertes JN, Anand P, Haggerty G, Kestenbaum M, Rosenblum GC (2015). The physician-patient working alliance and patient psychological attachment, adherence, outcome expectations, and satisfaction in a sample of rheumatology patients. Behav Med.

[27] Pflugeisen BM, Rebar S, Reedy A, Pierce R, Amoroso PJ (2016). Assessment of clinical trial participant patient satisfaction: a call to action. Trials.

[28] Kojima G (2018). Frailty defined by FRAIL Scale as a predictor of mortality: a systematic review and meta-analysis. J Am Med Dir Assoc.

[29] Morley JE, Malmstrom TK, Miller DK (2012). A simple frailty questionnaire (FRAIL) predicts outcomes in middle aged African Americans. J Nutr Health Aging.

[30] Woodend AK, Sherrard H, Fraser M, Stuewe L, Cheung T, Struthers C (2008). Telehome monitoring in patients with cardiac disease who are at high risk of readmission. Heart Lung.

[31] Chae YM, Lee JH, Ho SH, Kim HJ, Jun KH, Won JU (2001). Patient satisfaction with telemedicine in home health services for the elderly. Int J Med Inform.

[32] Uschner D, Schindler D, Hilgers R, Heussen N (2018). randomizeR: an R package for the assessment and implementation of randomization in clinical trials. J Stat Soft.

[33] Harris PA, Taylor R, Thielke R, Payne J, Gonzalez N, Conde JG (2009). Research electronic data capture (REDCap)--a metadata-driven methodology and workflow process for providing translational research informatics support. J Biomed Inform.

[34] (2021). Find clinical trials for you. Health Match.

[35] (2021). Join Us.

[36] Moore GF, Audrey S, Barker M, Bond L, Bonell C, Hardeman W, Moore L, O'Cathain A, Tinati T, Wight D, Baird J (2015). Process evaluation of complex interventions: Medical Research Council guidance. Br Med J.

[37] Bhaskar R (2011). Reclaiming Reality: A Critical Introduction to Contemporary Philosophy.

[38] Vasileiou K, Barnett J, Thorpe S, Young T (2018). Characterising and justifying sample size sufficiency in interview-based studies: systematic analysis of qualitative health research over a 15-year period. BMC Med Res Methodol.

[39] Dziura J, Post Lori A, Zhao Qing, Fu Zhixuan, Peduzzi Peter (2013). Strategies for dealing with missing data in clinical trials: from design to analysis. Yale J Biol Med.

[40] Tong A, Sainsbury P, Craig J (2007). Consolidated criteria for reporting qualitative research (COREQ): a 32-item checklist for interviews and focus groups. Int J Qual Health Care.

[41] Braun V, Clarke V (2006). Using thematic analysis in psychology. Qualitative Research in Psychology.

[42] Byrne D (1998). Complexity Theory and the Social Sciences: An Introduction.

[43] Gale NK, Heath G, Cameron E, Rashid S, Redwood S (2013). Using the framework method for the analysis of qualitative data in multi-disciplinary health research. BMC Med Res Methodol.

[44] Patton MQ (2014). Qualitative Research & Evaluation Methods: Integrating Theory and Practice, 4th Edition.

[45] Creswell JW, Hirose M (2019). Mixed methods and survey research in family medicine and community health. Fam Med Community Health.

[46] Leonardsen AL, Hardeland C, Helgesen AK, Grøndahl VA (2020). Patient experiences with technology enabled care across healthcare settings- a systematic review. BMC Health Serv Res.

[47] Chow CK, Redfern J, Hillis GS, Thakkar J, Santo K, Hackett ML, Jan S, Graves N, de Keizer L, Barry T, Bompoint S, Stepien S, Whittaker R, Rodgers A, Thiagalingam A (2015). Effect of lifestyle-focused text messaging on risk factor modification in patients with coronary heart disease: a randomized clinical trial. J Am Med Assoc.

[48] Somerville S, Somerville J, Croft P, Lewis M (2000). Atrial fibrillation: a comparison of methods to identify cases in general practice. Br J Gen Pract.

