# Supplementary material for: Patient-Led Mass Screening for Atrial Fibrillation in the Older Population Using Handheld Electrocardiographic Devices Integrated With a Clinician-Coordinated Remote Central Monitoring System: Protocol for a Randomized Controlled Trial and Process Evaluation
Source: JMIR Res Protoc. 2022 Feb 1;11(2):e34778. doi: 10.2196/34778 (PMC8848249; doi:10.2196/34778)
Supplement: Multimedia Appendix 1 [file resprot_v11i2e34778_app1.pdf]

Multimedia Appendix 1. Patient participant data collection at baseline, 6-month follow-up, and 12-month completion of the program and semistructured interview guide.

**Activity**	**Baseline**	**Weekdays**	**After 3 months**	**At 6th months or withdrawal or detection of AF**	**At 12th Month or withdrawal or detection of AF**
Socio-demographics	x				
Self-reported height and weight	x				
Single-lead ECG via mobile Kardia app		x			
Chronic health conditions (from the following list)<br>- Hypertension<br>- Coronary heart disease (angina, myocardial infarction, Percutaneous coronary intervention, coronary artery bypass surgery)<br>- Peripheral artery disease<br>- Aortic atherosclerosis<br>- Stroke<br>- Heart failure<br>- Diabetes<br>- Chronic obstructive pulmonary disease<br>- Asthma<br>- Arthritis<br>- Kidney disease<br>- Other	x				
Clarification of participants' use of medications.<br><br>Antihypertensive medications:<br>Beta-blocker<br>Calcium-channel blocker<br>ACE Inhibitor<br>Angiotensin receptor inhibitor<br>Diuretic<br><br>Statin<br>Antiplatelet<br>Anticoagulant<br><br>Diabetes medications:<br>Insulin<br>Others:	x			x	x
Stroke Risk Assessment (CHA2DS2-VASc Score)<br>- Cardiac failure (1 point):<br>- HTN (1 point):<br>- Age ≥ 75 y (2 point):<br>- Diabetes (1 point):<br>- Stroke (2 point):	x				

- Vascular disease (1 point): □ MI □ PAD □ aortic atherosclerosis<br>- Age 65-74 (1 point):<br>- Female sex (1 point):					
The FRAIL scale:<br>- **Fatigue**: "How much of the time during the past 4 weeks did you feel tired?" 1 = All of the time, 2 = Most of the time, 3 = Some of the time, 4 = A little of the time, 5 = None of the time. Responses of "1" or "2" are scored as 1 and all others as 0.<br>- **Resistance**: "By yourself and not using aids, do you have any difficulty walking up 10 steps without resting?" 1 = Yes, 0 = No.<br>- **Ambulation**: By yourself and not using aids, do you have any difficulty walking 1 km?" 1 = Yes, 0 = No.<br>- **Illnesses**: from the list of chronic conditions above, 0 - 4 = 0 and ≥5 conditions = 1<br>- **Loss of weight**: ask the participants their current weight and their weight in the previous year. If ≥ 5% loss of weight, scored as 1 and < 5% as 0. If they do not remember their weight, ask "Have you recently lost weight such that your clothing has become looser?", if Yes, scored as 1.	x				
Activities of Daily Living (ADLs): Do you have difficulty performing the following tasks on your own<br>- Eating<br>- Dressing<br>- Bathing<br>- Using the toilet<br>- Housekeeping<br>- Climbing stairs<br>- Walking<br>- Using transportation<br>- Managing medications<br>Answers are recorded as "yes" or "no"	x				
Social isolation: The Friendship Scale<br>In the past 4 weeks,<br>- I found it easy to get on with others: 1= almost always, 2= most of the time, 3= about half of the time, 4= occasionally, 5= not at all<br>- I had someone to share my feelings with: 1= almost always, 2= most of the time, 3= about half of the time, 4= occasionally, 5= not at all<br>- I found it easy to make contact with others: 1= almost always, 2= most of the time, 3= about half of the time, 4= occasionally, 5= not at all<br>- I felt lonely: 5= almost always, 4= most of the time, 3= about half of the time, 2= occasionally, 1= not at all<br>- I felt I was a burden to others: 5= almost always, 4= most of the time, 3= about half of the time, 2= occasionally, 1= not at all	x				

**Patient in-depth semi-structured interview**<br><br>**<u>Device-related questions:</u>**<br><br>1. What is your experience in using the AliveCor Kardia handheld ECG?<br><br>How did you find learning about how to use the device?<br><br>What did you find easy?<br>What did you find not so easy?<br><br>Do you have the adequate knowledge and skill to self-record an ECG at the beginning of the study (before receiving training from our research team)?<br><br>We sent a text message with a link to a video to show people or remind people how to record ECG. What was your impression of the video? Explain. (Did you understand it well)?<br><br>Do you involve family members to help you with recording ECG? Explain how you involve them.<br><br>Which family member helped you with the registration and training process (if any)?<br><br>Were our support and training to help you record an ECG adequate? (Explain why or why not).<br><br>What was it like using the AliveCor Kardia handheld ECG?<br><br>Did it work as you expected? (explain)<br><br>How satisfied were you with this device?<br><br>How easy was it to use?<br><br>What difficulties do you have?<br><br>2. How useful is this AliveCor Kardia handheld ECG in being able to monitor your heart rhythm?<br><br>Prompts:<br>Why do you think this is a useful way of monitoring heart rhythm?			x		

How well do you think this device would work for others in your community?<br>Which people would it suit best?<br><br>Why do you think it would benefit them? (How do you think others that you know would benefit from this device? Why/in what way?)<br><br>When do you prefer to record your ECG?<br><br>How do you value this method of screening in the community (rather than in a GP setting or hospital)?<br><br>*Explain more* (or *why* or *how is it valuable)*<br><br>3. If you had access to the device, will you continue using this device? Please explain your reasons.<br><br>4. Would you recommend this device to your family or friends? Please explain your reasons.<br><br>5. Have you used other mobile health devices?<br><br>Prompts:<br><br>Examples: Fitbit, Apple watch.<br><br>If yes, how comfortable are you in using mobile health devices generally?<br><br>What difficulties do you have?<br><br>**Program-specific questions:**<br><br>6. What is your overall experience in this screening program?<br><br>How comfortable are you with remote monitoring of your health?<br><br>What concerns would you have (if any)?<br><br>How comfortable are you with sending of your health data to a central monitor system and being accessed remotely by someone you don't know?<br><br>Please tell us more about your positive experience.					

Can you provide an example?<br><br>About your negative experience?<br><br>Can you provide an example?<br><br>What was it like being in this study and not having any in-person contact with the study team?<br><br>Has this program helped you established a daily routine that you find useful? (What is it? Explain).<br><br>Is there anything else about this screening program that you want to tell us?<br><br>How could the program be better?<br><br>If this remote health monitoring system were to be introduced, would you enrol in this type of program? (why/why not?)<br><br>If you were required to buy the ECG device to participate in the screening program:<br><br>(a) can you afford it?<br>(b) will you consider participating in the monitoring program? Why? (Reasons & suggestions)<br><br>7. For all participants: what is your experience with your access to health services? (Prompts: living condition, transport, need & availability of a support person).<br><br>Additional questions for patients diagnosed with AF: What action has your GP taken?<br><br>Have your GP added a medication to thin your blood?<br><br>How do you feel about the results of these actions?					
**Short survey question:**<br>How **satisfied** are you that your heart rhythm was being monitored in the past 6 months?<br>1 = Very satisfied, 2 = Satisfied, 3 = Somewhat satisfied, 4 = Not satisfied				x	
Clarification of participants' AF diagnosis in the control group				x	

In the past 6 months have you seen a General Practitioner? If yes, how many times?<br>In the past 6 months have you seen specialist doctors? If yes, how many times, and what specialist doctors?<br>In the past 6 months have you presented to Emergency or admitted to Hospital? If yes, please explain.				x	
**Usability questionnaire**:<br>How **easy** was the use of this device?<br>1 = Very difficult, 2 = Difficult, 3 = Average,<br>4 = Easy, 5 = Very easy<br><br>In term of the time taken to acquire an ECG tracing, how **efficient** was this device? 1 = Very inefficient, 2 = Inefficient,<br>3 = Average, 4 = Efficient, 5 = Very efficient<br><br>When you first received the device - How **anxious** were you in using this device?<br>How **anxious** are you currently in using this device? (In the past month)<br><br>1 = Extremely anxious, 2 = Very anxious, 3 = Moderately anxious, 4 = Slightly anxious, 5 = Not at all anxious<br><br>How comfortable were you in **sharing your personal information** and ECG with the research team?<br>1 = Not at all comfortable, 2 = Slightly comfortable, 3 = Generally comfortable, 4 = Very comfortable, 5 = Extremely comfortable<br><br>To what extent did the use of this device **restrict** your usual activities?<br>1 = Extremely restricted, 2 = Very restricted, 3 = Moderately restricted, 4 = Slightly restricted, 5 = Not at all restricted<br><br>How **confident** were you in your ability to use this device correctly?<br>1 = Not at all confident, 2 = Slightly confident, 3 = Generally confident, 4 = Very confident, 5 = Extremely confident<br><br>How **satisfied** were you with the use of this device?<br>1 = Not at all satisfied, 2 = Slightly Satisfied, 3 = Generally satisfied, 4 = Very satisfied, 5 = Extremely Satisfied<br><br>Do you agree that this **screening** method helps detect irregular heart rhythm in the community?					x

1 = Totally disagree, 2 = Disagree, 3 = Neutral, 4 = Agree,<br>5 = Strongly agree<br><br>Would you like to **continue usin**g this device if you have the choice?<br>1= no, 2= unlikely, 3= may be, 4= likely, 5= yes					
**Adverse events** occurred during the study duration: Death, Stroke/Transient Ischemic Attack ("temporary stroke"),<br>Clinically significant bleeds (bleeding that required medical treatment), Deep vein thrombosis/ pulmonary embolism ("blood clots"), Other cardiovascular disease (Other heart disease), Respiratory disease (Lung disease), Other neurological disease (Other disease in the nervous system),<br>Orthopaedic/musculoskeletal disease (disease in the bones or muscles), Fall, Gastroenterological disease (disease in the digestive system), Renal/urologic disease (disease in kidneys & urinary system), and other disease.			x	x	x
