# Supplementary material for: Patient-Led Mass Screening for Atrial Fibrillation in the Older Population Using Handheld Electrocardiographic Devices Integrated With a Clinician-Coordinated Remote Central Monitoring System: Protocol for a Randomized Controlled Trial and Process Evaluation
Source: JMIR Res Protoc. 2022 Feb 1;11(2):e34778. doi: 10.2196/34778 (PMC8848249; doi:10.2196/34778)
Supplement: Multimedia Appendix 2 [file resprot_v11i2e34778_app2.pdf]

**Multimedia Appendix 2. General practitioner data form and semistructured interview guide.**

**General Practitioner demographic data and role:**

**1. Which best describes you? (Please select one)**

GP with FRACGP ( )

GP with FACRRM ( )

GP with FRACGP and FACRRM ( )

GP Registrar ( )

Other (please specify) ( )____

**2. How long is it since you commenced working in general practice?**

less than 1 year ( )

1- 4 years ( )

5- 9 years ( )

10- 19 years ( )

20 years and more ( )

**3. Your gender:**

Male ( )

Female ( )

Rather not say ( )

**4. Approximately how many hours per week do you usually work in general practice?**

1-8 hours ( )

9-16 hours ( )

17-24 hours ( )

25-32 hours ( )

33-40 hours ( )

> 40 hours ( )

**5. What is the estimate of proportion of your patients aged 75 years and older?**

None ( )

1% to 10% ( )

11% to 20% ( )

21% to 30% ( )

31% to 40% ( )

41% to 50% ( )

51% to 60% ( )

61% to 70% ( )

71% to 80% ( )

81% to 90% ( )

91% to 100% ( )

**6. What is the postcode of your main practice? ______________________**

**7. What is your role in this research study? (Examples: facilitated recruitment of participants, managed participants with atrial fibrillation / arrhythmias etc.)**

______________________________________________________________________

**General Practitioner Survey**

(1) Do you perform opportunistic screening for atrial fibrillation at your practice?
Yes/ No

(2) Do you agree that this patient-led self-recording of single-lead ECG helps detect atrial fibrillation in the community?
1 = Totally disagree, 2 = Disagree, 3 = Neutral, 4 = Agree, 5 = Strongly agree

(3) Do you agree that this patient-led self-screening reduces time pressure in clinical practice?
1 = Totally disagree, 2 = Disagree, 3 = Neutral, 4 = Agree, 5 = Strongly agree

(4) Do you agree that this patient-led self-screening is a better alternative to an opportunistic screening in clinical practice?
1 = Totally disagree, 2 = Disagree, 3 = Neutral, 4 = Agree, 5 = Strongly agree

(5) How satisfied were you with this patient-led self-screening program?
1 = Not at all satisfied, 2 = Slightly Satisfied, 3 = Generally satisfied, 4 = Very satisfied, 5 = Extremely Satisfied

**General Practitioners in-depth semi-structured interview**

1. **About mobile health devices:**

What do you know about **mobile health devices generally**?

What mobile health devices do you use in your practice?

What are the positive aspects of the mobile health devices that you utilise?

What are the negative aspects?

What are your views about using **AliveCor handheld ECG for AF screening**?

What do you know about handheld ECG being used for AF screening?

Is the elderly group well suited to use handheld ECG for screening?
why/why not?

Are there population differences in this age group that need to be considered for use of handheld ECG devices (e.g. patients with language barrier)- explain

How confident are you with the accuracy of these devices?

How useful are these devices? Explain?

How do you want to use this device in your practice?

If you are not contemplating using handheld ECG device, please explain your reasons.

2. **About atrial fibrillation (AF) screening:**

What has been your experience diagnosing AF with your patients?

What about managing AF?

**In general, what are your views about AF screening?**

Prompts:

What experience do you have with this type of screening before this AF screening study?
(e.g. when/where did you use it)

(if experience) Can you describe your experience (e.g. what worked, did not work?)

**What are your views of AF screening in people aged 75 years and older**?

Prompts:

Is this age group well suited to this type of screening in your view?
why/why not?

Are there population differences in this age group that need to be considered (e.g. indigenous population, patients with language barrier)- explain

**Are you aware of any guidelines regarding AF screening?** Such as the Australian AF Guidelines established by the National Heart Foundation of Australia (NHFA) and Cardiac Society of Australia and New Zealand (CSANZ).

What are your views of the Guidelines?

How can you apply the Guidelines in your practice?

**What are your views of patient-led self-recording of single-lead ECG as a method to screen for AF in this research study?**

Prompts:

Where did it work best/where was it least effective?

With which patients?

Why do you think there were differences?

What are the barriers for this type of screening?

Are there barriers in the general practice setting such as the infrastructure, facility, and resources in general practice?

What are the enablers?

**What cardiologist services do you access in managing new cases of AF?**

Prompts:

Was it your local cardiologist or our cardiology team?

What was your experience in using the cardiologist service?

**How would you improve the screening program?** (prompts: alternative screening device/ method, GP-patient interactive process)

**How could this type of patient-led self-screening fit in your practice workflow?**

What is your role in this type of patient-led self-screening?
